# Psychological interventions countering misinformation in social media: A scoping review

**DOI:** 10.3389/fpsyt.2022.974782

**Published:** 2023-01-05

**Authors:** Paweł Gwiaździński, Aleksander B. Gundersen, Michal Piksa, Izabela Krysińska, Jonas R. Kunst, Karolina Noworyta, Agata Olejniuk, Mikołaj Morzy, Rafal Rygula, Tomi Wójtowicz, Jan Piasecki

**Affiliations:** ^1^Department of Philosophy and Bioethics, Faculty of Health Sciences, Jagiellonian University Medical College, Kraków, Poland; ^2^Consciousness Lab, Institute of Psychology, Jagiellonian University, Kraków, Poland; ^3^Department of Psychology, University of Oslo, Oslo, Norway; ^4^Affective Cognitive Neuroscience Laboratory, Department of Pharmacology, Maj Institute of Pharmacology of the Polish Academy of Sciences, Kraków, Poland; ^5^Poznań University of Technology, Poznań, Poland

**Keywords:** misinformation, social media, scoping review, systematic review, psychological interventions, Facebook, Twitter, Reddit

## Abstract

**Introduction:**

The rise of social media users and the explosive growth in misinformation shared across social media platforms have become a serious threat to democratic discourse and public health. The mentioned implications have increased the demand for misinformation detection and intervention. To contribute to this challenge, we are presenting a systematic scoping review of psychological interventions countering misinformation in social media. The review was conducted to (i) identify and map evidence on psychological interventions countering misinformation, (ii) compare the viability of the interventions on social media, and (iii) provide guidelines for the development of effective interventions.

**Methods:**

A systematic search in three bibliographic databases (PubMed, Embase, and Scopus) and additional searches in Google Scholar and reference lists were conducted.

**Results:**

3,561 records were identified, 75 of which met the eligibility criteria for the inclusion in the final review. The psychological interventions identified during the review can be classified into three categories distinguished by Kozyreva et al.: Boosting, Technocognition, and Nudging, and then into 15 types within these. Most of the studied interventions were not implemented and tested in a real social media environment but under strictly controlled settings or online crowdsourcing platforms. The presented feasibility assessment of implementation insights expressed qualitatively and with numerical scoring could guide the development of future interventions that can be successfully implemented on social media platforms.

**Discussion:**

The review provides the basis for further research on psychological interventions counteracting misinformation. Future research on interventions should aim to combine effective Technocognition and Nudging in the user experience of online services.

**Systematic review registration:**

[https://figshare.com/], identifier [https://doi.org/10.6084/m9.figshare.14649432.v2].

## 1. Introduction

The world has witnessed an unprecedented spread of misinformation in recent years (1–3). Waves of misinformation are responsible for diminishing social trust in public health agencies, sowing social discord, encouraging, and strengthening xenophobic, homophobic, and nationalistic stances, and undermining popular confidence in the benevolence of democratic institutions (4–6). Misinformation is an umbrella term which encompasses several similar phenomena: intentional and unintentional spreading of false information, disseminating urban legends, sharing fake news, unverified information, and rumor, as well as crowdturfing, spamming, trolling, and propagating hate speech, or being involved in cyberbullying (7, 9–12). Detection of fake news and rumors is attracting significant attention from the research community (13). Similarly, many studies aim to understand the psychological factors that contribute to the individuals’ increased susceptibility to misinformation. Given this scientific effort, a comparison of various psychological interventions (for the definition of “psychological intervention,” see section “2 Materials and methods”) to immunize individuals against misinformation is of both theoretical and practical importance.

A psychological intervention that protects online users against misinformation can take many forms. The most straightforward intervention is manipulating the user interface by adding warnings (14), tags (15), credibility scores (16), or fact-checks (17). Another possibility is to display information in the social context (e.g., by showing indicators of peer acceptance or rejection of information) (16). Another solution is to inoculate users by teaching them to recognize misinformation (18) or improving their media (19) and science literacy (20) or engaging users using gamification (21). The question remains: which type, and modality of psychological intervention is most likely to succeed in a given context? This scoping review provides an overview of existing psychological interventions designed to fight the spread of misinformation, compare them, and provide design guidelines to maximize their effectiveness. While the underlying psychological mechanisms of misinformation are beyond the scope of this manuscript, we hope it can serve as a useful starting point for future analysis in this respect.

We followed the PRISMA Extension for Scoping Reviews [PRISMA-ScR (22)] to identify recent research on psychological interventions countering misinformation spread. The initial pool of studies identified via database search or manual citation search via Google Scholar consisted of 4279 publications. After removing duplicates, we screened 3,561 publications by titles and abstracts. Finally, the application of the eligibility criteria reduced the pool of studies to 75 publications selected for information extraction. While reviewing the papers, we focused on types of interventions, not types of studies, as the latter would lean more toward the goals of a meta-analysis rather than a scoping review.

Three findings stand out as the main result of the scoping review. We identified five major types of study designs and assessed the efficacy of psychological interventions that were based on them. We further developed a typology of 15 distinct subtypes nested within three broader classifications of psychological interventions. We also designed an intervention viability assessment score survey (see Table 3 in Supplementary material) to evaluate the possible reach and overall cost of their implementation on the existing social media (Facebook, Twitter, etc.), and we applied this assessment score to all the studies included in this scoping review. The results revealed the two most promising types in terms of viability of psychological interventions: Message from a trusted organization and Source rating.

**TABLE 1:** Glossary of key terms used in current study, (see Figure 2).

Types of study designs		
		• **Ecological** – Study design that evaluates the influence of environmental factors on individual behavior and mental health (23) • **Non-ecological** – Study design that does not account for the influence of environmental factors on individual behavior and mental health • **Mimical** – Study design that employs stimuli closely resembling a social media UX design while still being heavily controlled and performed in a lab or online setting • **Game** – Study design that tests gamified approaches to fighting misinformation in social media • **Mixed methods** – Study design that uses multiple types of study designs
**Categories and types of psychological interventions**		
	• **Boosting** – Cognitive interventions and tools that aim to foster people’s cognitive and motivational competencies (e.g., simple rules for online reasoning) (24)	
		• Inoculation – Inoculation theory is a framework originating from social psychology. It posits that it is possible to preemptively confer psychological resistance against (malicious) persuasion attempts (18, 25). It is a kind of deliberate action aimed at improving the latent ability to spot misinformation techniques, as opposed to just individual instances of misinformation (18, 21, 26, 27). Usually, it is done by exposing participants to misinformation in order to teach them its structures and mechanisms • Fact-checking – Based on confronting misinformation online with factual information from credible sources, which is done, for instance, by webpages whose goal is debunking misinformation, such as snopes.com • Media literacy – Educational intervention aimed at increasing the subject’s knowledge about misinformation risks in social media and training the ability to recognize misinformation • Science literacy – Educational intervention aimed at increasing the subject’s knowledge about scientific conduct, discerning good science from bad, and training to recognize scientific misinformation • Public pledge to the truth – Pro-truth pledge is an initiative that tries to incentivize misinformation protecting behaviors by encouraging subjects to make a public vow to commit to truth-oriented behaviors and protect facts and civility • Anti-cyberbullying video – Educational videos designed to sensitize subjects to the issues regarding cyberbullying • **Technocognition** – Cognitively inspired technological interventions in information architectures (e.g., introducing friction in the sharing of offensive material) (28) • UX manipulation – Utilizing manipulations to user’s interface and ways they interact with social media to fight misinformation online. • Deliberation – The process of carefully considering the content before sharing, rating, or commenting on it. These kinds of interventions are meant to incentivize subjects to take time to deliberately process content. • Source rating – Based on grading systems used to evaluate the credibility of an information source that is then displayed to users.
	• **Nudging** – Behavioral interventions in the choice architecture that alter people’s behavior in a predictable way (e.g., automatic [default] privacy-respecting settings) (29)	
		• Warning – Based on notifying the subject beforehand that the online content they are about to consume might contain misinformation • Tagging – Aimed at detecting and tagging misinformative content, usually with some visual sign or notification • Social correction – An intervention enacted by a group, demanding appropriate behavior from an individual. On the contrary, in normative and empathy nudges, the subject is messaged privately by a single person (or a bot) • Correction – Aimed at correcting inaccurate information (mostly in the scientific domain). Correction is usually embedded in the content, for instance, at the beginning or at the end of an article • Empathy nudge – An intervention in which another person’s pressure elicits a more empathetic stance on the subject • Message from a trusted organization – Based on sending corrective, fact-checking messages from a widely trusted organization’s account

**TABLE 2:** Inclusion criteria for scoping review.

The paper focuses on some form of misinformation
The paper is empirical
The paper addresses the issues of misinformation in the social media context
The paper was published after 2004
The paper proposes a psychological intervention
The paper is peer-reviewed
The paper is published in English
The paper presents experimental manipulations aimed at reducing susceptibility to misinformation in social media

**TABLE 3:** Patient, Intervention, Comparison, and Outcome (PICO) search strategy disambiguation.

P – patient	(“disinformation” OR “misinformation” OR “fake news” OR “conspiracy theor*” OR “urban legend*” OR “rumor*” OR “hate speech” OR “cyberbullying” OR “fake science” OR “mislead*” OR “fake source*” OR “propagand*”) AND (“social media” OR “facebook” OR “instagram” OR “twitter” OR “tiktok” OR “youtube” OR “messenger” OR “whatsapp” OR “telegram” OR “internet” OR “media” OR “blog*” OR “reddit” OR “4chan”)
I – intervention	(“intervent*” OR “tag*” OR “factcheck*” OR “false-tag” OR “refutation” OR “correct*” OR “retraction” OR “flag*” OR “headline*” OR “counter*” OR “rated false” OR “disrupted” OR “questionnaire*” OR “survey*” OR “interview*” OR “focus group*” OR “case stud*” OR “observ*” OR “experiment*” OR “qualitative” OR “quantitative” OR “mixed method*” OR “experiment*”)
C – comparison	(“view*” OR “experienc*” OR “opinion*” OR “attitude*” OR “perce*” OR “belie*” OR “judge*” OR “feel*” OR “know*” OR “understand*” OR “assess*” OR “expect*” OR “tenden*”)
O – outcome	(“share*” OR “verify” OR “follo*” OR “unfollo*” OR “subscrib*” OR “unsubscrib*” OR “click*” OR “induc*” OR “trust*” OR “distrust*” OR “check*” OR “reduc*” OR “judge*” OR “inferenc*” OR “correct*” OR “reflect*” OR “reliance” OR “resist*” OR “back-fire” OR “influe*” OR “like”)

## 2. Materials and methods

**FIGURE 1:**
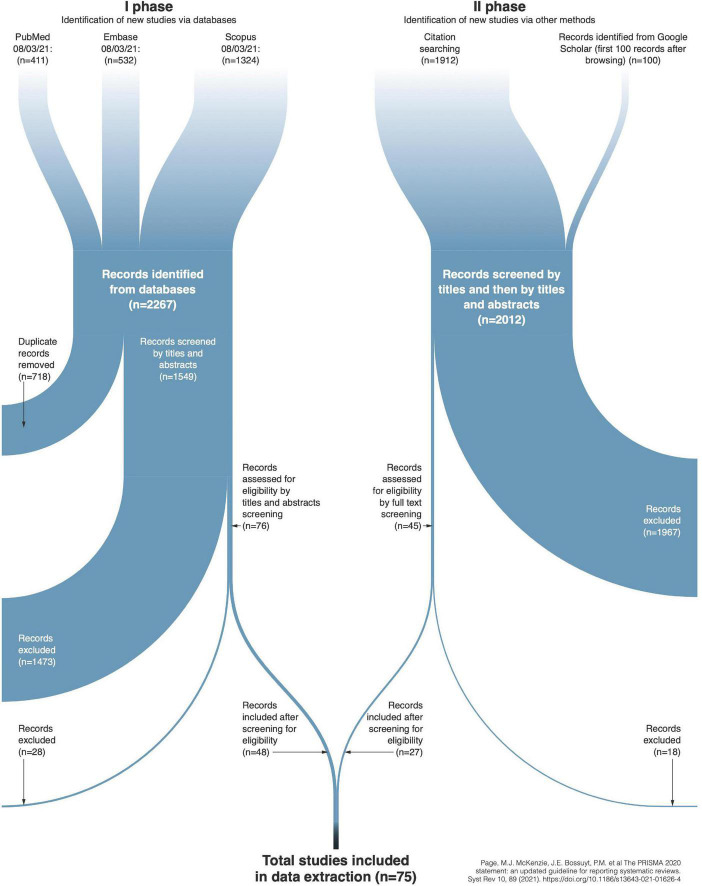
PRISMA workflow of scoping review.

This scoping review is reported according to the PRISMA-ScR (see Figure 1) reporting criteria for scoping reviews (see Table 2 in Supplementary material). The protocol was pre-registered and published in the Jagiellonian University Repository (30).

### 2.1. Eligibility criteria

The process of developing the eligibility criteria was inspired by both the classical approach to systematic reviews (31) and by more modern approaches, focused on the qualitative methods of reviews (32). However, PICO is more sensitive than modified strategies and it is recommended for systematic reviews (33). Thus, the eligibility criteria were based on the PICO (Population, Intervention, Comparison, and Outcome) components and the specification of the types of studies such as publication status and language. After adjusting the PICO scheme to the requirements of the scoping review, we formulated the eligibility criteria in terms of the PIO (Population, Intervention, “Outcome) scheme” (Table 2).

• **Population:** In order to be included in the review, a study had to focus on one of the forms of misinformation (i.e., the spread of false information, urban legends, fake news, fake science) or address the issues of misinformation in social media (e.g., Facebook, Twitter, Instagram, or pairings of those). In defining misinformation, we utilize Wu et al.’s definition (7) which lists kinds of misinformation as: intentional and unintentional spreading of false information, disseminating urban legends, sharing fake news, unverified information, and rumor, as well as crowdturfing [the term means: leveraging human-powered crowdsourcing platforms to spread malicious URLs in social media and manipulate search engines, ultimately degrading the quality of online information and threatening the usefulness of these systems (8)], spamming, trolling, and propagating hate speech, or being involved in cyberbullying (7, 9–12). This definition allowed us to operationalize the “Population” part of the search query.
• **Intervention:** Interventions eligible for the review must be psychological interventions that counter misinformation. A psychological intervention is understood here as an intervention and/or experimental manipulation that targets psychological, intermediary, or cognitive processes or actual behavior (23). An example of a psychological intervention might be asking subjects to pause to consider why a headline is true or false before sharing. An intervention is not psychological when it targets, e.g., either biochemical functions of a body (e.g., pharmacological intervention) or the functions of a computer/phone (e.g., computer processing information on a phone). A compatible definition of the intervention considered in this review is the one that can be found in the APA Dictionary of Psychology: “strategies and processes designed to measure the change in a situation or individual after a systematic modification (diet, therapeutic technique) has been imposed or to measure the effects of one type of intervention program as compared to those of another program. Experimentation is the most common type of intervention research, but clinical trials and qualitative studies may also be used.” (23). As such, experimental manipulations to reduce susceptibility to misinformation in social media will be included in this review. Interventions eligible for the review cannot be speculative or impossible to employ in a social media environment: for instance, interventions requiring the involvement of highly trained specialists should be excluded.
• **Outcome:** To be included in the review, a study also must be empirical, i.e., present primary data obtained through a qualitative and/or quantitative research methodology, which implies that reviews, meta-analyses, theoretical, or other non-empirical papers have to be excluded.
• **Additional criteria:** The scoping review included only peer-reviewed studies published after 2004. The choice of the date is deliberate as it corresponds to the launching of Facebook, the oldest modern-scale social network. In addition, we consider only peer-reviewed studies published in English.

When screening studies to fulfill the eligibility criteria, whenever relevant information was missing from studies, the reviewers attempted to contact the study authors to obtain the required information.

### 2.2. Study selection

The search strategy protocol was developed based on the Joanna Briggs Institute recommendations that assume a three-step strategy: preliminary search, the first phase, and the second phase (31).

*Preliminary search*: The preliminary search was aimed at selecting the keywords and index terms for constructing a search query that drives the prime search. For this purpose, the authors searched three databases: Scopus, PubMed, and later, on Google Scholar from 01/01/2004 to present with a set of keywords. The search was limited to English language studies as per the eligibility criteria. The search was conducted on 03/12/2020. The authors manually analyzed the retrieved studies to identify candidate search terms by looking at the terminology used and the subject indexing in the records. The final query was constructed using a PICO-style approach. Table 3 presents search terms related to each component of the PICO framework. In the preliminary searches, we also tested different bases (APA PsycInfo, Sage, Google Scholar); the final list of three data bases (PubMed, Embase, and Scopus) was chosen for the first search because they returned a large number of records, enabled transparent and replicable searches, as well as enabled the use of Boolean operators.

The final query (Table 3 and see Supplementary material) was formulated according to the PICO formula: (P) AND (I) AND (C) AND (O).

The search query was validated by testing whether it could identify the two known relevant studies (34, 35) as part of the search strategy development process.

*First phase*: In the first phase, the search query was issued to three databases: PubMed, Embase, and Scopus. The query was issued on 08/03/2021.

*Second phase*: In the second phase, all references cited in the studies meeting the criteria returned from the first phase were screened for inclusion concerning the eligibility criteria. In addition, a simplified search query (Query 2) was issued to the Google Scholar search engine on 28/07/2021.

The date coverages and query execution dates are given in Table 4. The final search results were exported into the EndNote tool. A detailed description of the search strategy can be found in the Table 3 in Supplementary material.

**TABLE 4:** Query execution dates.

Stage	Database	Coverage	Query execution date
Preliminary search	PubMed	NA – 02/12/2020	03/12/2020
	Scopus	NA – 02/12/2020	03/12/2020
	Google Scholar	NA – 14/07/2021	31/07/2021
First phase	PubMed	2004 – 08/03/2021	08/03/2021
	Scopus	2004 – 08/03/2021	08/03/2021
	Embase	2004 – 08/03/2021	08/03/2021
Second phase	Google Scholar	2004 – 28/07/2021	28/07/2021

### 2.3. Data extraction

Eligible studies were equally assigned to pairs of contributors for data extraction. Each contributor collected the data independently and discussed inconsistencies until consensus was reached within the pair. In case of unreported or inaccessible data, the contributors tried to obtain this information from the study’s authors.

The following data items have been extracted from each study included in the review:

• **Bibliographic data:** authors, publication venue, year of publication, funding, type of publication, conflict of interest, corresponding author affiliation,
• **Study metadata:** inclusion and exclusion criteria for participants, risk of bias,
• **Cohort data:** demographic data describing the population undergoing psychological intervention,
• **Study design:** type of misinformation addressed by a study, study design and study methodology, social media being studied,
• **Interventions and outcome:** description of the intervention, the time it takes for an intervention to be successful, the viability of the intervention application, and eventual follow-up study outcomes (to establish whether an intervention has left persisting effects among users).

All the collected details of studies are included in Table 1 and Data Sheet in Supplementary material. For the detailed PRISMA Scoping Review Workflow see Figure 1.

### 2.4. Data synthesis

Qualitative data concerning study design, intervention outcomes, and types of interventions was synthetized using inductive methods inspired by the constant comparative method: similar study designs, intervention outcomes, and types of interventions were joined into one category (36, 37). The inductive process was conducted by four coders who agreed to the final version of the qualitative categories. Moreover, after distinguishing 15 different types of psychological intervention, we used a broad categorization developed by Kozyreva et al. and we sorted our 15 types into those three general intervention categories (38).

The intervention assessment score (IAS) was a measure developed to merely supplement the narrative synthesis of the paper, and its methodology is based on the grounded theory and abductive method (39). In this line of work, inter-rater reliability (IRR) is not something that is desired. As McDonald et al. (40) point out for grounded theories, codes are “merely” an interim product that supports the development of a theory, not a final result that requires testing. We treated the rating codes of interventions as an ethnography performed by an interdisciplinary team of experts, and differing scores are something that is expected here by design, as it is impossible to take the preliminary experiences out of the ethnographer, as Barkhuus and Rossitto point out (41).

## 3. Results

### 3.1. Study selection

The selection consisted of two phases. The first phase involved searching PubMed, Embase, and Scopus databases, which resulted in the identification of 2,267 records. Deduplication excluded 718 records, and screening, according to the inclusion criteria (see section “2.1 Eligibility criteria”), rejected 1,501 records. Thus, the first phase resulted in the selection of 48 eligible records. The second phase included 1,912 publications cited in the eligible records identified in the first phase. The second phase also included 100 papers from a Supplementary Google Scholar search. Screening, according to the inclusion criteria, rejected 1,985 records. Thus, the second phase resulted in the selection of 27 eligible records. In total, the selection process yielded 75 eligible records (for details, see Table 1 in Supplementary material).

### 3.2. Types of study design

We have identified five distinct types of study designs: ecological, non-ecological, mimical, game, and mixed methods. *Ecological* studies were conducted within the social media environment, and participants were often unaware of either the study’s objective or the fact of being studied (42). *Non-ecological* studies were usually conducted in a heavily controlled laboratory setting (34). Alternatively, non-ecological studies were performed online using carefully prepared interfaces, often bearing little resemblance to the social media user experience design [UX design, e.g., (20)]. *Mimical* studies employed stimuli closely resembling social media UX design, e.g., scrolling a website resembling the Facebook timeline, while being conducted in a heavily controlled environment (35). Several studies tested *gamified* approaches to fighting misinformation in social media (43). Finally, *mixed methods* encompass studies using multiple approaches and experiments within one study (44). Non-ecological and mimical studies are the dominant type of study designs. Ecological studies, which provide insight into the more “natural” behavior of users of social networks, are still scarce (Figure 3).

**FIGURE 2:**
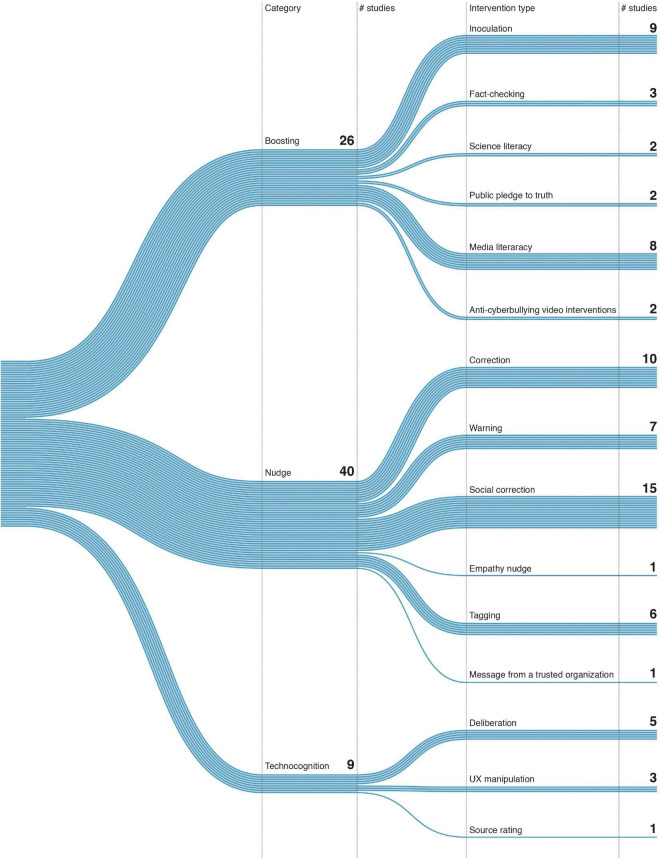
Misinformation psychological interventions typology based on Kozyreva et al. (38).

**FIGURE 3:**
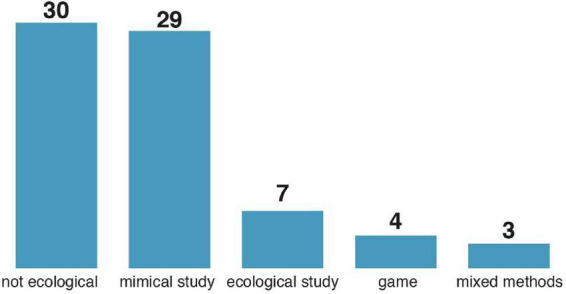
Study design types distribution in the sample.

### 3.3. Types of psychological interventions

We used a general typology of psychological interventions proposed by Kozyreva et al. (38), dividing interventions into three categories and 15 types (Figure 2). The categories refer to different evidence-based behavioral and cognitive interventions that can be applied to the digital world. Technocognition refers to interventions that change how users experience and consume content in social media, for instance, by introducing some friction in the way user shares information. This includes UX manipulation, Deliberation, Source rating. Boosting interventions are cognitive interventions and tools that aim to foster people’s cognitive and motivational competencies (24) and include the following: Inoculation, Fact-checking, Science literacy, Public pledge to the truth, Media literacy, Anti-cyberbullying video. Nudging includes behavioral interventions in the choice architecture that alter people’s behavior in a predictable way (e.g., default privacy-respecting settings (29)). They include: Correction, Warning, Social correction, Empathy nudge, Tagging, Message from a trusted organization.

### 3.4. Publications by year

**FIGURE 4:**
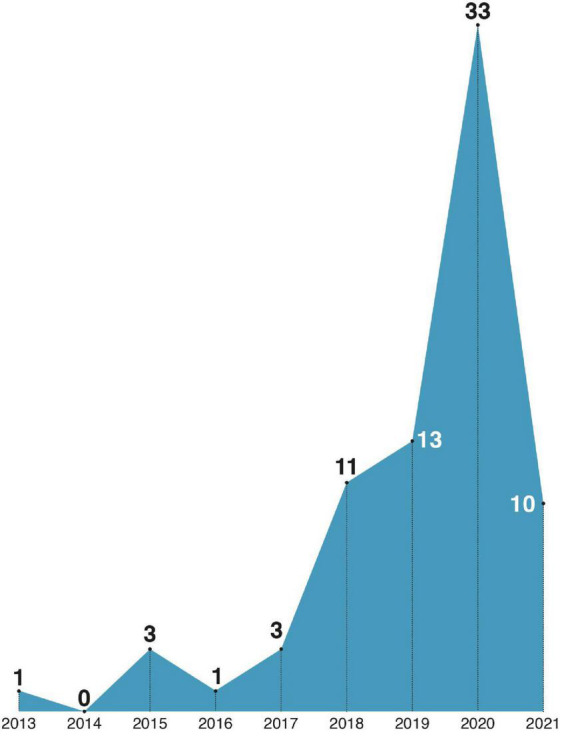
Social media misinformation year of publications distribution in the sample.

We did not find any studies on psychological interventions counteracting the spread of misinformation in social networks prior to 2013. We find this surprising as the topic of “fake news” was present in both public and scientific discourse already in the first decade of the century. This is perhaps caused by the fact that the public awareness of the problem is still growing. In 2016, the term “post-truth” was included in the Oxford English Dictionary and chosen as Word of the Year (45). The narrative of people living in the “post-truth era” gained momentum at that point. We are also observing a rapid increase in the number of studies published in the years following this event (see Figure 4). In our opinion, this trend yields evidence of the urgency of fighting the misinformation circulating in online social networks.

### 3.5. Psychological intervention outcomes

**FIGURE 5:**
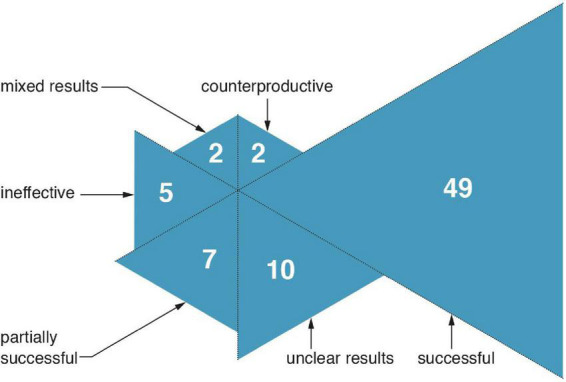
Author’s intervention test conclusion in the sample.

For each study, we extracted the description of the outcome and conclusions drawn by the authors of the study regarding the successfulness of the implemented intervention. We identified six possible outcomes: successful, partially successful, mixed result, unclear result, ineffective, and counterproductive (i.e., an intervention increased the susceptibility to misinformation) (see Figure 5). The majority of studies (46) included in the review reported a successful outcome of the intervention tests. For 10 studies, the authors concluded that the results were unclear, and more research was needed to evaluate the effectiveness of the given intervention. The interventions which were successful in general but either could be further improved, or whose positive effect was weak, are classified as “partially successful”; we found 7 of these. Finally, the authors of 5 studies did not find any evidence of a positive effect of an intervention, and two interventions were deemed counterproductive.

### 3.6. Psychological intervention assessment score

**FIGURE 6:**
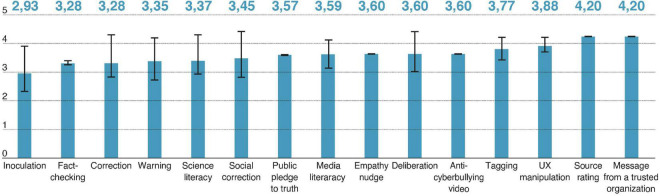
Mean viability score by intervention type.

To gain further insight into the viability and practical use of psychological interventions, we computed an intervention assessment score (IAS) for each study included in the review (see Figure 6). IAS was designed not to score effectiveness, but viability, which effectiveness is just part of. This score was based on ratings on a 5-point Likert scale (for details, see Table 3 in Supplementary material). Each item was designed to rate, as follows: the successfulness of the intervention, the technical ease of implementation, the amount of resources needed for intervention to be implemented, whether it requires motivated participants, whether it requires massive change to the way social media currently work. The rating was performed by the raters: PG, JP, MP, and AG. The team of raters was interdisciplinary and included researchers with different views on each rated item. This approach was intentional, as opposed to traditional expert rating, where it is assumed that there is only one good rating for each item. As the subject of misinformation-countering interventions is complex, there might be varying views on the viability of different aspects of such interventions. For instance, in Item 2, the raters were asked to evaluate whether “This intervention seems to be technically easy to implement in social media, based on your knowledge.” For a rater with a cognitive science and programming background, this statement might be interpreted as “easy to code and implement,” whereas a rater with a psychological background might rate this item having the users’ perspectives and their underlaying psychological mechanisms in mind. We think that both views are valid and by averaging these differing ratings, we obtain a score that is more general rather than limited to a specific field, as it encompasses broader aspects of the interventions. Taking the above into account, the inter-rater reliability scores such as Cohen’s kappa would be meaningless in this case, as they require experts from heterogenous fields, trained to interpret material in the same manner.

### 3.7. Social media and topics

**FIGURE 7:**
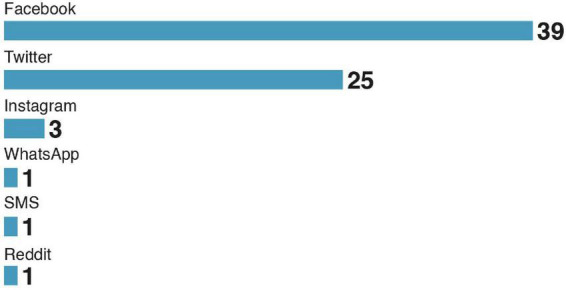
Number of papers published by type of social media studied in the sample.

**FIGURE 8:**
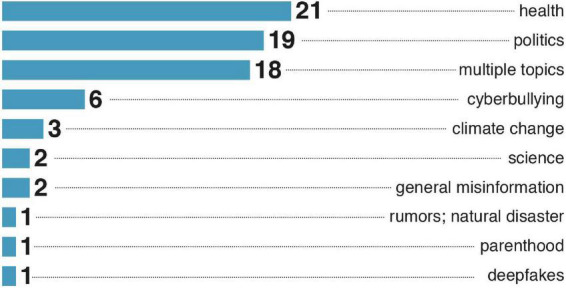
Number of publications by misinformation types in the sample.

Facebook and Twitter are the primary targets for psychological intervention (see Figure 7). Interestingly, we did not find studies on psychological interventions in video-based social networks (TikTok, YouTube). Possibly, the form of the text-based social media made it easier to implement psychological interventions. Figure 8 presents the distribution of topics considered for psychological intervention. We found health and politics to be the primary areas of misinformation research.

### 3.8. Demographics

The mean age of the reviewed study participants was 35.05 years. The age of the participants in this field is surprisingly low, as it has been suggested that older adults are more vulnerable to misinformation and are more often responsible for spreading it (47). Judging from the corresponding author location, we can say that by far most research on misinformation in social media has been conducted in the USA (43 papers). The UK and Germany are a far second (six papers each), followed by the Netherlands (five papers). In the analyzed sample, three teams emerge as those most published. The most published team is led by E.K. Vraga and published ten papers on the effectiveness of social correction and media literacy promotion. The second most published team is Roozenbeek-van der Linden’s team with six publications. The team has a very concentrated focus on the theory of inoculation and game interventions. Finally, there is a team led by Pennycook, which published five papers that test the effects of deliberation, accuracy nudge, and tagging.

## 4. Discussion

The purpose of the scoping review was to provide an overview of the existing psychological interventions designed to combat the spread of misinformation in social media and to compare them with respect to their viability. We classified the psychological interventions into three broader categories after Kozyreva et al.: Boosting, Technocognition, Nudge, out of which four types were rated as the most viable: Source rating, Message from a trusted organization, UX manipulation, and Tagging. In those intervention types, the subject is not required to be a highly motivated fact-checker and, depending on the design, they can encompass a wide variety of misinformation aspects [for instance, they can incorporate a non-binary approach to the truth of a given article (15)]. Those intervention types have already found their use in social media, e.g., via browser extensions.^1^ Technicognition and Nudging interventions can usually be automated with the help of chatbots, and they have been proven effective (44, 48, 49), as opposed to Boosting interventions, which require vast resources and highly motivated participants, therefore, they were rated as least viable (they might be most effective in the long run, however). It is also important to note that all the studies included in the scoping review are relatively new. Half of the papers have been published in the last 3 years, which seems to coincide with the need for misinformation-related research due to the events that are taking place in Western Europe and the USA, both in terms of the political scene and the COVID-19 pandemic.

One important limitation of the results of the scoping review is the fact that the reviewed studies under-represent older participants, in particular, people older than 65 years. Another limitation is the almost exclusive focus on text-based social media such as Facebook and Twitter, excluding the newer, more visually focused media, such as TikTok, You Tube, and Instagram. Unfortunately, the review does not allow us to conclude that the types of psychological interventions that are successful for more traditional social media would be equally successful for more image-based or video-based media. On the contrary, introducing corrections or peer and social pressure markers may be much more difficult in the latter case, if the psychological intervention is performed via text (e.g., adding a link to a fact-checking website). However, a study testing the effectiveness of psychological inoculation by means of short YouTube clips which has been published recently, after the conclusion of our review, shows some promising results (46).

Our review provides the basis for further research on psychological interventions counteracting the spread of misinformation. Future research on interventions should aim to combine effective Technocognition with various types of Nudging, e.g., seamlessly immersing normative, peer, and social pressure indicators in the user experience of online services. Future interventions should also focus on areas culturally different from Western Europe and the US where most of the studies have been conducted. Cultural differences and class divisions play an important role in misinformation susceptibility. Users originating from vulnerable or excluded groups interact with misinformation differently than cohorts studied in the scoping review (50, 51). Diversification of research perspectives may be essential when designing psychological interventions for these users. Moreover, scoping reviews and, even more importantly, systematic reviews with meta-analysis measuring the effectiveness of interventions should be conducted to catch up with continuously published new studies (27, 52–55) and to supplement the results of traditional reviews (56) which have been recently published on this issue.(57, 58).

### 4.1. Risk of bias

In terms of selection bias, two factors should be considered: restraining searches to a limited number of databases and the rapidly growing number of studies on mitigating social media misinformation published after conducting searches (27, 52–55). In order to mitigate the risk of selection bias, the authors conducted a supplementary search consisting of an additional Google Scholar search and a bibliographic search. To reduce the risk of rejecting relevant studies, all the records retrieved from the searches were screened against the eligibility criteria independently by two reviewers. It is also worth stressing that the design choices behind the IAS, while encompassing the broad spectrum of views on the matter, do not allow using any statistical tools to exclude the possibility of bias.

## Author contributions

PG and JP: conceptualization, methodology, search strategy, validation, data acquisition, and supervision. PG, JP, MP, and AG: data extraction and investigation. PG: data curation and writing – original draft. JP, MM, IK, TW, MP, AG, RR, JK, and KN: writing – review and editing. JP: supervision. JP, MM, JK, and RR: project administration and funding acquisition. All authors have read and agreed to the published version of the manuscript.

## Footnotes

1
https://mediabiasfactcheck.com/appsextensions/
