# Supplementary material for: Psychological interventions countering misinformation in social media: A scoping review
Source: Front Psychiatry. 2023 Jan 5;13:974782. doi: 10.3389/fpsyt.2022.974782 (PMC9849948; doi:10.3389/fpsyt.2022.974782)
Supplement: Supplementary file 1 [file Data_Sheet_1.docx]

Supplementary Material

# Search queries

## 1st phase query

Query 1 : (("disinformation" OR "misinformation" OR "fake news" OR "conspiracy theor*" OR "urban legend*" OR "rumor*" OR "hate speech" OR "cyberbullying" OR "fake science" OR "mislead*" OR "fake source*" OR "propagand*") AND ("social media" OR "facebook" OR "instagram" OR "twitter" OR "tiktok" OR "youtube" OR "messenger" OR "whatsapp" OR "telegram" OR "internet" OR "media" OR "blog*" OR "reddit" OR "4chan")) AND ("intervent*" OR "tag*" OR "factcheck*" OR "false-tag" OR "refutation" OR "correct*" OR "retraction" OR "flag*" OR "headline*" OR "counter*" OR "rated false" OR "disrupted" OR "questionnaire*" OR "survey*" OR "interview*" OR "focus group*" OR "case stud*" OR "observ*" OR "experiment*" OR "qualitative" OR "quantitative" OR "mixed method*" OR "experiment*") AND ("view*" OR "experienc*" OR "opinion*" OR "attitude*" OR "perce*" OR "belie*" OR "judge*" OR "feel*" OR "know*" OR "understand*" OR "assess*" OR "expect*" OR "tenden*") AND ("share*" OR "verify" OR "follo*" OR "unfollo*" OR "subscrib*" OR "unsubscrib*" OR "click*" OR "induc*" OR "trust*" OR "distrust*" OR "check*" OR "reduc*" OR "judge*" OR "inferenc*" OR "correct*" OR "reflect*" OR "reliance" OR "resist*" OR "backfire" OR "influe*" OR "like")

## 2nd phase query

Query 2: (“disinformation” OR “misinformation” OR “fake news” OR “conspiracy theory” OR “fake science”) AND (“intervention”) AND (“social media” OR “facebook” OR “instagram” OR “twitter” OR “tiktok” OR “youtube” OR “messenger” OR “whatsapp” OR “telegram” OR “internet” OR “media” OR “blog” OR “reddit” OR “4chan”)

# Intervention Assessment Score Survey

In order to assess the efficacy of psychological interventions reported in the studies included in the review, in parallel with data extraction the contributors completed an intervention assessment score survey using the following form:

**Intervention assessment score survey**

Please rate the intervention employed in this paper on a 1-5 scale, where 1 means ‘strongly disagree’ and 5 means ‘strongly agree’.

1. This intervention was proven in the paper to be successful (qualitative analysis).

Strongly Disagree 1------2------3------4------5 Strongly Agree

2. This intervention seems to be technically easy to implement in social media, based on your knowledge.

Strongly Disagree 1------2------3------4------5 Strongly Agree

3. This intervention does not require vast technological or financial resources to be implemented.

Strongly Disagree 1------2------3------4------5 Strongly Agree

4. This intervention does not require highly motivated participants.

Strongly Disagree 1------2------3------4------5 Strongly Agree

5. This intervention does not require big changes in the way social media UX currently works.

Strongly Disagree 1------2------3------4------5 Strongly Agree

**3. List of included articles – compare table 1.**

1. Bhuiyan MM, Zhang K, Vick K, Horning MA, Mitra T. Feedreflect. Companion of the 2018 ACM Conference on Computer Supported Cooperative Work and Social Computing. 2018;
2. Bode L, Tully M, Vraga EK. Do the right thing: Tone may not affect correction of misinformation on social media. Harvard Kennedy School Misinformation Review. 2020;
3. Kim A, Moravec PL, Dennis AR. Combating fake news on social media with source ratings: The effects of user and expert reputation ratings. Journal of Management Information Systems. 2019;36(3):931–68.
4. Kluck JP, Schaewitz L, Krämer NC. Doubters are more convincing than advocates. the impact of user comments and ratings on credibility perceptions of false news stories on social media. Studies in Communication and Media. 2019;8(4):446–70.
5. Gao M, Xiao Z, Karahalios K, Fu W-T. To label or not to label. Proceedings of the ACM on Human-Computer Interaction. 2018;2(CSCW):1–16.
6. Munger K. Tweetment effects on the tweeted: Experimentally reducing racist harassment. Political Behavior. 2016;39(3):629–49.
7. Naab TK, Heinbach D, Ziegele M, Grasberger M-T. Comments and credibility: How critical user comments decrease perceived news article credibility. Journalism Studies. 2020;21(6):783–801.
8. Roozenbeek J, van der Linden S. Fake news game confers psychological resistance against online misinformation. Palgrave Communications. 2019;5(1).
9. Basol M, Roozenbeek J, Van der Linden S. Good news about bad news: Gamified inoculation boosts confidence and cognitive immunity against fake news. Journal of Cognition. 2020;3(1):2.
10. Pennycook G, Epstein Z, Mosleh M, Arechar AA, Eckles D, Rand DG. Shifting attention to accuracy can reduce misinformation online. Nature. 2021;592(7855):590–5.
11. Roozenbeek J, van der Linden S. Breaking harmony square: A game that “inoculates” against political misinformation. Harvard Kennedy School Misinformation Review. 2020;
12. Moravec PL, Kim A, Dennis AR. Appealing to Sense and Sensibility: System 1 and System 2 Interventions for Fake News on Social Media. Information Systems Research. 2020;31(3).
13. Ozturk, P., Li, H., & Sakamoto, Y. (2015, January). Combating rumor spread on social media: The effectiveness of refutation and warning. In 2015 48th Hawaii international conference on system sciences (pp. 2406-2414). IEEE.
14. Porter E, Wood TJ, Kirby D. Sex Trafficking, Russian Infiltration, Birth Certificates, and Pedophilia: A Survey Experiment Correcting Fake News. Journal of Experimental Political Science. 2018;5(2):159–64
15. Roozenbeek J, van der Linden S, Nygren T. Prebunking interventions based on “inoculation” theory can reduce susceptibility to misinformation across cultures. The Harvard Kennedy School Misinformation Review. 2020;1(2).
16. Ross B, Jung A, Heisel J, Stieglitz S. Fake News on Social Media: The (In)Effectiveness of Warning Messages. In: Proceedings of the 39th International Conference on Information Systems (ICIS 2018). San Francisco, USA: Association for Information Systems; 2018.
17. Tanaka Y, Sakamoto Y, Matsuka T. Toward a social-technological system that inactivates false rumors through the critical thinking of crowds. 2013 46th Hawaii International Conference on System Sciences. 2013;
18. Tully M, Vraga EK, Bode L. Designing and Testing News Literacy Messages for Social Media. Mass Communication and Society. 2019;23(1):22–46.
19. van der Meer TA, Jin Y. Seeking Formula for Misinformation Treatment in Public Health Crises: The Effects of Corrective Information Type and Source. Health Commun. 2020;35(5):560–75.
20. Vraga EK, Kim S.C., Cook J. Testing logic-based and humor-based corrections for Science, health, and political misinformation on social media. Journal of Broadcasting & Electronic Media. 2019;63(3):393–414.
21. Bode L, Vraga EK. See something, say something: Correction of global health misinformation on social media. Health Communication. 2018;33(9):1131–40.
22. Roozenbeek J, Maertens R, McClanahan W, van der Linden S. Disentangling item and testing effects in inoculation research on online misinformation: Solomon revisited. Educational and Psychological Measurement. 2021;81(2):340–62.
23. Fazio L. Pausing to consider why a headline is true or false can help reduce the sharing of false news. Harvard Kennedy School Misinformation Review. 2020;
24. Maertens R, Roozenbeek J, Basol M, van der Linden S. Long-term effectiveness of inoculation against misinformation: Three longitudinal experiments. Journal of Experimental Psychology: Applied. 2021;27(1):1–16.
25. Tsipursky G, Votta F, Mulick JA. A psychological approach to promoting truth in politics: The pro-truth pledge. Journal of Social and Political Psychology. 2018;6(2):271–90.
26. Hazan R. TIPS TO STOP FAKE NEWS Assessing the Effectiveness of Different Digital Literacy Interventions Fit for Social Media [thesis]. [Princeton]: Princeton University Archives ; 2021. p. 1–81.
27. Kang R. Accuracy in Spotting Misinformation about COVID-19: A Pilot Intervention and the Role of Political Affiliation [thesis]. Unpublished; 2021. p. 1–59.
28. Bago B, Rand DG, Pennycook G. Fake news, fast and slow: Deliberation reduces belief in false (but not true) news headlines. Journal of Experimental Psychology: General. 2020;149(8):1608–13.
29. Bilewicz M, Tempska P, Leliwa G, Dowgiałło M, Tańska M, Urbaniak R, et al. Artificial Intelligence Against Hate: Intervention reducing verbal aggression in the Social Network Environment. Aggressive Behavior. 2021;47(3):260–6.
30. Bowles J, Larreguy H, Liu S. Countering misinformation via WhatsApp: Preliminary evidence from the COVID-19 pandemic in Zimbabwe. PLOS ONE. 2020;15(10).
31. Brashier NM, Pennycook G, Berinsky AJ, Rand DG. Timing matters when correcting fake news. Proceedings of the National Academy of Sciences. 2020;118(5).
32. Valdez CA, Ziefle M. Believability of News : Understanding Users Perceptions of Fake News and the Effectiveness of Fact-Checking Badges. In: Proceedings of the 20th Congress of the International Ergonomics Association (IEA 2018) : Volume V: Human Simulation and Virtual Environments, Work With Computing Systems (WWCS). 2018. p. 469–77.
33. Chen X, Sin SCJ, Theng YL, Lee CS. Deterring the spread of misinformation on social network sites: A Social Cognitive theory‐guided intervention. Proceedings of the Association for Information Science and Technology. 2015;52(1):1–4.
34. Chua AYK, Banerjee S. Intentions to trust and share online health rumors: An experiment with medical professionals. Computers in Human Behavior. 2018;87:1–9.
35. Clayton K, Blair S, Busam JA, Forstner S, Glance J, Green G, et al. Real solutions for fake news? measuring the effectiveness of general warnings and fact-check tags in reducing belief in false stories on social media. Political Behavior. 2019;42(4):1073–95.
36. Clever L, Assenmacher D, Müller K, Seiler MV, Riehle DM, Preuss M, et al. Fakeyou! - A gamified approach for building and evaluating resilience against fake news. Disinformation in Open Online Media. 2020;:218–32.
37. Cook J, Lewandowsky S, Ecker UK. Neutralizing misinformation through inoculation: Exposing misleading argumentation techniques reduces their influence. PLOS ONE. 2017;12(5).
38. Doane AN, Kelley ML, Pearson MR. Reducing cyberbullying: A theory of reasoned action-based video prevention program for college students. Aggressive Behavior. 2015;42(2):136–46.
39. Ecker UK, O'Reilly Z, Reid JS, Chang EP. The effectiveness of short‐format refutational fact‐checks. British Journal of Psychology. 2019;111(1):36–54.
40. Featherstone JD, Zhang J. Feeling angry: The effects of vaccine misinformation and refutational messages on negative emotions and vaccination attitude. Journal of Health Communication. 2020;25(9):692–702.
41. Figl K, Kießling S, Rank C, Vakulenko S. Fake News Flags, Cognitive Dissonance, and the Believability of Fake News Flags, Cognitive Dissonance, and the Believability of Social Media Posts Social Media Posts . In: ICIS 2019 Proceedings 27. 2019. p. 692–702.
42. Gesser-Edelsburg A, Diamant A, Hijazi R, Mesch GS. Correcting misinformation by health organizations during measles outbreaks: A controlled experiment. PLOS ONE. 2018;13(12).
43. Guess AM, Lerner M, Lyons B, Montgomery JM, Nyhan B, Reifler J, et al. A digital media literacy intervention increases discernment between mainstream and false news in the United States and India. Proceedings of the National Academy of Sciences. 2020;117(27):15536–45.
44. Hameleers M. Separating truth from lies: Comparing the effects of news media literacy interventions and fact-checkers in response to political misinformation in the US and Netherlands. Information, Communication & Society. 2020;25(1):110–26.
45. Hameleers M, van der Meer TG. Misinformation and polarization in a high-choice media environment: How effective are political fact-checkers? Communication Research. 2020;47(2):227–50.
46. Hameleers M, Powell TE, Van Der Meer TGLA, Bos L. A picture paints a thousand lies? the effects and mechanisms of multimodal disinformation and rebuttals disseminated via social media. Political Communication. 2020;37(2):281–301.
47. Huang Y, Wang W. When a story contradicts: Correcting health misinformation on social media through different message formats and mechanisms. Information, Communication & Society. 2020;:1–18.
48. Iacobucci S, De Cicco R, Michetti F, Palumbo R, Pagliaro S. Deepfakes Unmasked: The effects of information priming and bullshit receptivity on deepfake recognition and sharing intention. Cyberpsychology, Behavior, and Social Networking. 2021;24(3):194–202.
49. Garrett RK, Poulsen S. Flagging facebook falsehoods: Self-identified humor warnings outperform fact checker and Peer Warnings. Journal of Computer-Mediated Communication. 2019;24(5):240–58.
50. Kim A, Dennis A. Says who?: How news presentation format influences perceived believability and the engagement level of social media users. Proceedings of the 51st Hawaii International Conference on System Sciences. 2018;
51. Kim JW, Masullo Chen G. Exploring the influence of comment tone and content in response to misinformation in social media news. Journalism Practice. 2020;15(4):456–70.
52. Kim SC, Vraga EK, Cook J. An eye tracking approach to understanding misinformation and correction strategies on social media: The mediating role of attention and credibility to reduce HPV vaccine misperceptions. Health Communication. 2020;36(13):1687–96.
53. Kirchner J, Reuter C. Countering fake news: A Comparison of Possible SolutionsRegarding User Acceptance and Effectiveness. Proceedings of the ACM on Human-Computer Interaction. 2020;4(CSCW2):1–27.
54. Lee J. The effect of web add-on correction and narrative correction on belief in misinformation depending on motivations for using social media. Behaviour & Information Technology. 2020;:1–15.
55. Leung AN, Fung DC-L, Farver JAM. A cyberbullying intervention for Hong Kong chinese college students. Applied Research in Quality of Life. 2017;13(4):1037–53
56. Lutzke L, Drummond C, Slovic P, Árvai J. Priming critical thinking: Simple interventions limit the influence of fake news about climate change on Facebook. Global Environmental Change. 2019;58:101964.
57. Martel C, Mosleh M, Rand DG. You’re definitely wrong, maybe: Correction style has minimal effect on corrections of misinformation online. Media and Communication. 2021;9(1):120–33.
58. Masullo GM, Kim J. Exploring “angry” and “like” reactions on uncivil Facebook comments that correct misinformation in the news. Digital Journalism. 2020;9(8):1103–22.
59. Mena P. Cleaning up social media: The effect of warning labels on likelihood of sharing false news on Facebook. Policy & Internet. 2019;12(2):165–83.
60. Miškolci J, Kováčová L, Rigová E. Countering hate speech on Facebook: The case of the roma minority in Slovakia. Social Science Computer Review. 2018;38(2):128–46.
61. Moravec PL, Kim A, Dennis AR. Appealing to Sense and Sensibility: System 1 and System 2 Interventions for Fake News on Social Media. Information Systems Research. 2020;31(3).
62. Pennycook G, Bear A, Collins ET, Rand DG. The implied truth effect: Attaching warnings to a subset of fake news headlines increases perceived accuracy of headlines without warnings. Management Science. 2020;66(11):4944–57.
63. Pennycook G, McPhetres J, Zhang Y, Lu JG, Rand DG. Fighting covid-19 misinformation on social media: Experimental evidence for a scalable accuracy-nudge intervention.
64. Smith CN, Seitz HH. Correcting misinformation about neuroscience via social media. Science Communication. 2019;41(6):790–819.
65. Tanaka Y, Hirayama R. Exposure to countering messages online: Alleviating or strengthening false belief? Cyberpsychology, Behavior, and Social Networking. 2019;22(11):742–6.
66. Taylor SH, DiFranzo D, Choi YH, Sannon S, Bazarova NN. Accountability and empathy by design: Encouraging Bystander Intervention to Cyberbullying on Social Media. Proceedings of the ACM on Human-Computer Interaction. 2019;3(CSCW):1–26.
67. Tsipursky G, Votta F, Roose KM. Fighting fake news and post-truth politics with behavioral science: The pro-truth pledge. Behavior and Social Issues. 2017;27(1):47–70.
68. Tully M, Vraga EK, Bode L. Designing and Testing News Literacy Messages for Social Media. Mass Communication and Society. 2019;23(1):22–46.
69. van Stekelenburg A, Schaap G, Veling H, Buijzen M. Investigating and improving the accuracy of US citizens’ beliefs about the covid-19 pandemic: Longitudinal Survey Study. Journal of Medical Internet Research. 2021;23(1).
70. Vraga EK, Bode L, Tully M. Creating news literacy messages to enhance expert corrections of misinformation on Twitter. Communication Research. 2020;49(2):245–67.
71. Vraga EK, Kim SC, Cook J, Bode L. Testing the effectiveness of correction placement and type on Instagram. The International Journal of Press/Politics. 2020;25(4):632–52.
72. Vraga EK, Bode L. I do not believe you: How providing a source corrects health misperceptions across social media platforms. Information, Communication & Society. 2017;21(10):1337–53.
73. Vraga EK, Bode L. Addressing COVID-19 misinformation on social media preemptively and responsively. Emerging Infectious Diseases. 2021;27(2):396–403.
74. Zhang J, Featherstone JD, Calabrese C, Wojcieszak M. Effects of fact-checking social media vaccine misinformation on attitudes toward vaccines. Preventive Medicine. 2021;145:106408.
75. Zhao W. Misinformation correction across social media platforms. 2019 International Conference on Computational Science and Computational Intelligence (CSCI). 2019;
