# Supplementary material for: Psychological interventions countering misinformation in social media: A scoping review
Source: Front Psychiatry. 2023 Jan 5;13:974782. doi: 10.3389/fpsyt.2022.974782 (PMC9849948; doi:10.3389/fpsyt.2022.974782)
Supplement: Supplementary file 4 [file Table_2.docx]

## ID Names

**of authors (APA)**

1. Bhuiyan, M. M., et al.

## Year

2018

## Misinfor- mation kind

multiple topics

## Codes for ecological study

ecological study

## Description

**of intervention**

UX manipulation

## Social media studied

**Author’s conclusion**

successful intervention

## Viability

technically feasible

**Intervention assessment score**

# 3,9

1. Bode L., et al.

2020

multiple topics

mimical study

Social correction

successful intervention

proven feasibility, requires resources

**4,0**

1. Kim, A., et al.

2019

politics

not ecological

Tagging

successful intervention

technically feasible

**4,2**

1. Kluck, J. P., et al.
2. Gao, M., et al.

2019

2018

politics

politics

ecological study

not ecological

Social correction

UX manipulation

successful intervention

counterproductive results

technically feasible

technically feasible

**3,8**

# 2,4

1. Munger, K.

2016

cyberbullying

ecological study

Social correction

unclear results

proven feasibility

**3,9**

1. Naab, T.K., et al.

2020

multiple topics

mimical study

Social correction

mixed results

ineffective; Specific: a voluntary action

**3,5**

1. Roozenbeek, J., et al.

2019

multiple topics

not ecological

Inoculation

successful intervention

technically feasible

**3,9**

1. Basol, M., et al.

2020

multiple topics

not ecological

Inoculation

successful intervention

Specific: requires more research

**3,7**

1. Pennycook, G., et al.

2021

politics

mixed methods

Deliberation

successful intervention

proven feasibility

**4,4**

1. Roozenbeek, J., et al.

2020

multiple topics

not ecological

Inoculation

successful intervention

technically feasible

**3,8**

1. Moravec, P. L., et al.
2. Ozturk, P., et al.

2020

2015

parenthood

health

not ecological

not ecological

Tagging

Deliberation

partially successful intervention

successful intervention

proven feasibility

technically feasible

**3,7**

# 3,0

1. Porter, E., et al.

2018

politics

unclear

Correction

successful

NA intervention

requires vast resources

**2,8**

1. Roozenbeek, J., et al.

2020

multiple topics

game

Inoculation

successful intervention

requires motivation

**2,5**

1. Ross, B., et al.
2. Tanaka, Y., et al.
3. Tully, M., et al.

2018

2013

2019

multiple topics

rumors; natural disaster

health

mimical study

mimical study

mimical study

Warning

Social correction

Media literacy

ineffective intervention

successful intervention

successful intervention

requires vast resources: limited effectiveness

technically feasible

proven feasibility

**2,7**

# 2,8

**3,7**

1. van der Meer,

T. A., & Jin, Y.

2020

health

mimical study

Correction

successful intervention

technically feasible

**3,0**

1. Vraga, E. K., et al.

2019

multiple topics

mimical study

Correction

successful intervention

technically feasible

**2,8**

1. Bode, L., & Vraga, E. K.
2. Roozenbeek, J., & van der Linden, S.
3. Fazio, L. K.

2018

2020

2020

health

multiple topics

politics

mimical study

game

mimical study

Social correction

Inoculation

Deliberation

successful intervention

successful intervention

successful intervention

technically feasible

requires motivation

proven feasibility

**2,8**

# 2,3

**3,5**

1. Maertens, R., et al.

2021

multiple topics

game

Inoculation

successful intervention

requires motivation

**2,6**

1. Tsipursky, et al.


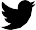

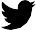

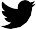

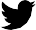

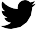

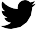

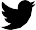

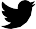

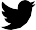

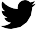

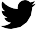

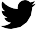

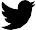

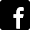

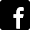

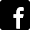

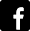

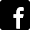

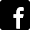

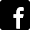

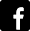

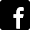

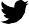

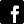

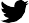

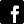


2018

multiple topics

ecological study

Public pledge to truth

successful intervention

requires motivation

**3,6**
