# Supplementary material for: Psychological interventions countering misinformation in social media: A scoping review
Source: Front Psychiatry. 2023 Jan 5;13:974782. doi: 10.3389/fpsyt.2022.974782 (PMC9849948; doi:10.3389/fpsyt.2022.974782)
Supplement: Supplementary file 5 [file Table_3.docx]

## ID Names

**of authors (APA)**

1. Hazan, R.

## Year

2021

## Misinfor- mation kind

politics

## Codes for ecological study

unclear

## Description

**of intervention**

Media literacy

## Social media studied

NA

## Author’s conclusion

mixed results

## Viability

proven feasibility

**Intervention assessment score**

# 3,4

1. Kang, R,

2021

health

game

Inoculation

successful intervention

requires motivation

**2,5**

1. Bago B., et al.

2020

politics

not ecological

Deliberation

successful

NA intervention

proven feasibility; Specific: requires user motivation

**3,7**

1. Bilewicz M., et al. 2020

cyberbullying

ecological study

Social correction

successful intervention

proven feasibility

**4,4**

1. Bowles, J., et al.

2020

health

mixed methods

Warning

successful intervention

proven feasibility

**4,2**

1. Brashier, N. M., et al.

2020

politics

not ecological

Correction

successful

NA intervention

proven feasibility

**4,2**

1. Valdez, A. C. & Ziefle, M.

2018

politics

mimical study

Tagging

unclear results

technically feasible

**3,4**

1. Chen, X., et al.

2015

general misinformation

mimical study

Media literacy

successful intervention

requires motivation

**3,9**

1. Chua, A. Y. K. & Banerjee, S.

2018

health

not ecological

Warning

ineffective

NA intervention

requires vast resources

**2,8**

1. Clayton, K., et al.

2019

politics

mimical study

Warning

successful intervention

proven feasibility

**4,2**

1. Clever, L., et al.

2020

multiple topics

not ecological

Inoculation

unclear results

NA

requires motivation

**2,7**

1. Cook, J., et al.

2017

climate change not

ecological

Inoculation

partially

NA successful intervention

technically feasible

**3,0**

1. Doane, A. N., et al.

2015

cyberbullying

not ecological

Anti-cyberbullying video interventions

successful

NA intervention

requires motivation

**3,6**

1. Ecker U. K. H, et al.

2020

multiple topics

not ecological

Tagging

successful intervention

technically feasible

**3,9**

1. Featherstone,

J. D., & Zhang, J.

2020

health

not ecological

Correction

N/A

successful intervention

proven feasibility

**3,6**

1. Figl, K., et al.

2019

multiple topics

not ecological

Tagging

successful intervention

technically feasible

**3,6**

1. Gesser-

Edelsburg, A., et al.

1. Guess, A. M., et al.

2018

2020

health

multiple topics

not ecological

not ecological

Science literacy

Media literacy

partially successful intervention

unclear results

NA

requires motivation

ineffective

**4,3**

# 3,8

1. Michael

Hameleers

2020

politics

not ecological

Media literacy

successful intervention

proven feasibility

**4,0**

1. Hameleers, M., et al.

2020

politics

not ecological

Fact-checking

successful

NA intervention

proven feasibility

**3,4**

1. Hameleers, M., et al.
2. Huang, Y.,

& Wang, W.,

2020

2020

general misinformation

health

not ecological

not ecological

Correction

Correctionsuccessful intervention

unclear results

proven feasibility

proven feasibility

**4,3**

# 3,8

1. Iacobucci, S., et al.

2021

deepfakes

not ecological

Media literacy

successful intervention

proven feasibility

**4,1**

1. Garrett, R. K., & Poulsen, S.


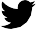

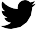

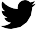

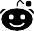

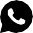

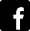

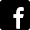

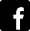

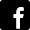

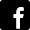

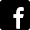

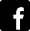

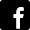

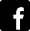

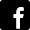


1. Kim, A.,

& Dennis, A.R.

2019

2018

politics

politics

not ecological

not ecological

Tagging

Source rating

partially successful intervention

successful intervention

technically feasible; Specif.: requires more research

technically feasible

**4,2**

**4,2**
