# Supplementary material for: Psychological interventions countering misinformation in social media: A scoping review
Source: Front Psychiatry. 2023 Jan 5;13:974782. doi: 10.3389/fpsyt.2022.974782 (PMC9849948; doi:10.3389/fpsyt.2022.974782)
Supplement: Supplementary file 6 [file Table_4.docx]

| **ID** | **Names** | **Year** | **Misinfor-** | **Codes for** | **Description** | **Social** | **Author’s** | **Viability** | **Intervention** |
| --- | --- | --- | --- | --- | --- | --- | --- | --- | --- |
|  | **of authors** |  | **mation** | **ecological** | **of intervention** | **media** | **conclusion** |  | **assessment** |
|  | **(APA)** |  | **kind** | **study** |  | **studied** |  |  | **score** |

1. Kim, J. W.,

& Masullo Chen, G.

1. Kim, S. C., et al.

2020

2020

politics

health

not ecological

not ecological

Social correction

Correction

successful intervention

unclear results

voluntary action

proven feasibility

**4,4**

# 3,5

1. Kirchner, J., & Reuter, C.

2020

multiple topics

not ecological

UX manipulation

successful intervention

successful intervention

**4,2**

1. Lee, J.

2020

health

ecological study

Correction

successful intervention

requires motivation

**3,1**

1. Leung, A. N., et al.

2017

cyberbullying

mimical study

Anti-cyberbullying video interventions

successful intervention

requires motivation

**3,6**

1. Lutzke, L., et al.
2. Martel, C., et al.

2019

2021

climate change

politics

mimical study

mimical study

Media literacy

Social correction

partially successful intervention

unclear results

NA

technically feasible

requires vast resources

**3,2**

# 3,2

1. Masullo,

G. M., & Kim, J.

1. Mena, P.

2021

2020

health; immigration policy

politics

mimical study

mimical study

Social correction

Warning

unclear results

successful intervention

requires vast resources

proven feasibility

**3,2**

# 3,5

1. Miskolci, J., et al.

2020

cyberbullying

ecological study

Social correction

unclear results

technically feasible

**3,1**

1. Moravec,

P. L., et al.

2020

multiple topics

mimical study

Warning

successful intervention

requires vast resources

**3,4**

1. Pennycook, G., et al.

2020

politics

mimical study

Warning

counterproductive results

technically feasible

**3,3**

1. Pennycook, G., et al.

2020

health

mimical study

Deliberation

successful intervention

proven feasibility

**4,0**

1. Smith, C. N., & Seitz, H. H.

2019

science

mimical study

Fact-checking

successful intervention

requires vast resources

**3,2**

1. Tanaka, Y.,

& Hirayama, R.

1. Taylor, S. H., et al.
2. Tsipursky, G., et al.

2019

2019

2018

science

cyberbullying

NA

mimical study

mixed methods; mocked UX experiment

ecological study

Social correction

Empathy nudge

Public pledge to truth

partially successful intervention

partially successful intervention

successful intervention

requires vast resources

proven feasibility

requires motivation

**2,8**

# 3,6

**3,6**

1. Tully, M., et al.

2020

health

mimical study

Social correction

unclear results

technically feasible

**3,2**

1. van Stekelenburg, 2021 et al.

health

not ecological

Science literacy

ineffective

NA intervention

technically feasible

**2,9**

1. Vraga, E. K., & Bode, L.

2021

health

mimical study

Social correction

ineffective intervention

requires vast resources

**3,6**

1. Vraga, E. K., & Bode, L.

2017

health

mimical study

Social correction

successful intervention

technically feasible

**3,6**

1. Vraga, E. K., et al.

2020

health

mimical study

Media literacy

ineffective intervention

technically feasible

**3,1**

1. Vraga, E. K., et al.

2020

climate change

mimical study

Correction

successful intervention

proven feasibility

**3,1**

1. Zhang, J., et al.

2021

health

mimical study

Fact-checking

successful intervention

requires vast resources

**3,3**

1. Zhao, W.


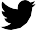

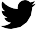

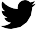

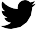

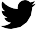

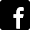

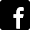

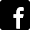

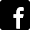

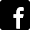

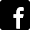

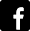

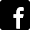

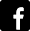

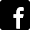

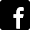

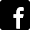

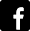

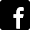

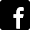

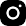

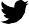

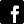

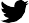

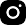

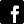

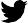

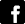


2019

health

mimical study

Correction

successful intervention

requires vast resources: limited effectiveness

**3,0**
